# Serum concentration of dickkopf-related protein 1 (DKK1) in psoriatic arthritis in the context of bone remodelling

**DOI:** 10.1007/s00296-023-05452-w

**Published:** 2023-09-26

**Authors:** Grzegorz Biedroń, Marcin Czepiel, Maciej Siedlar, Mariusz Korkosz

**Affiliations:** 1 Department of Rheumatology and Immunology, Jagiellonian University Medical College, Jakubowskiego 2, Krakow, Poland; 2 Department of Clinical Immunology, Institute of Pediatrics, Jagiellonian University Medical College, Krakow, Poland

**Keywords:** DKK1, Dickkopf-1, Dickkopf-related protein 1, Psoriatic arthritis, PsA;, Spondyloarthropathy, Bone remodelling

## Abstract

Psoriatic arthritis (PsA) is a chronic inflammatory disease, characterised by the pathological occurrence of two opposite phenomena—osteoresorption and osteogenesis. Dickkopf-related protein 1 (DKK1) which inhibits the Wingless protein (Wnt) signalling pathway has been shown to be a master regulator of bone remodeling in inflammatory rheumatic diseases. However, the exact relationship between DKK1 serum level and bone remodelling is not clear. The goal of this study is to review state-of-the-art knowledge on the association of serum DKK1 with a bone remodelling in PsA. The MEDLINE-PubMed, EMBASE, Scopus, Web of Science and DOAJ databases were searched for appropriate papers. The English terms: ‘DKK1’, ‘Dickkopf-1’ ‘Dickkopf related protein 1’, ‘psoriatic arthritis’ and ‘PsA’ were used for search purposes. Eight original articles and two reviews were identified up to August 2023. In four out of 8 discussed studies DKK1 serum level was higher in PsA patients than in healthy controls [Dalbeth, *p* < 0.01; Diani, *p* < 0.001; Chung, *p* < 0.01; Abd el Hamid, *p* < 0.001)], it was comparable in another (Daousiss, *p = *0.430) and was lower in two (Fassio2017, *p* < 0.05; Fassio2019, *p* < 0.05). In one study, the comparative groups included patients with axial spondyloarthritis, where DKK1 serum levels were lower in PsA groups [Jadon, peripheral PsA, *p = *0.01]. The true relative serum concentration of DKK1 in PsA, as well as its influence on osteogenesis and osteoresorption, is still equivocal. Further studies on this matter with consistent and stringent methodology are warranted.

## Background

Psoriatic arthritis (PsA) is a chronic inflammatory disease that belongs to the family of spondyloarthropathies (SpA). It may affect axial and peripheral joints, as well as entheses, digits (dactylitis) and nails. In most cases, it is also associated with skin psoriasis. The pathological bone remodelling in PsA consists of two opposite phenomena—osteoresorption and osteogenesis, which are often present simultaneously in one individual. Excessive bone formation is the hallmark of PsA and manifests as osteoproliferation, entesophyte and syndesmophyte (or parasyndesmophyte) formation and sacroiliac joint ankylosis, leading to the impairment of joint function and progressing disability of the affected patient [1–3].

One of the most important molecules responsible for bone remodelling is Dickkopf-related protein 1 (DKK1) which inhibits the Wingless protein (Wnt) signalling pathway promoting new bone formation [4]. DKK1 has been shown to be a master regulator of bone remodelling in inflammatory rheumatic diseases, including SpA [5].

The goal of this study is to review state-of-the-art knowledge on the association of serum DKK1 with a bone remodelling in PsA.

## Search methodology

The aim of the search process was to determine the number of studies with measured DKK1 serum level in PsA subjects. The MEDLINE-PubMed, EMBASE, Scopus, Web of Science and DOAJ databases were searched for appropriate papers. The English terms: ‘DKK1’, ‘Dickkopf-1’ ‘Dickkopf related protein 1’, ‘psoriatic arthritis’ and ‘PsA’ were used for retrieving search purposes. The inclusion criteria encompassed papers written in English, published in scientific journals (as letters to the editor, original articles, reviews, meta-analyses), in which the measured values of DKK1 serum level in PsA cohorts were available (in text, figures and/or tables). The diagnosis of PsA needed to be established on the basis of Moll and Wright criteria or Classification Criteria for Psoriatic Arthritis (CASPAR) or reliably confirmed by the authors of the study (when meeting the Moll and Wright or CASPAR criteria was not literally mentioned). The papers not written in English, published only as abstracts (without full text) or preliminary versions were excluded. Studies were carefully analysed for fullfilling the criteria. Eight original articles and two reviews meeting the requirements were identified in August 2023.

## Bone remodelling in PsA

Bone remodelling is a physiological process, which consists of two opposite phenomena—osteogenesis and osteoresorption. The proper balance between them is necessary to maintain bone mineral density and bone tissue homeostasis. These processes involve complex interactions between different cells, hormones, cytokines, growth factors and signalling pathways [6–8]. When bone formation and bone resorption are uncoupled, osteoproliferation, syndesmophyte, parasyndesmophyte or enthesophyte formation and bone erosion may occur [9].

Bone remodelling in PsA is driven by cytokines, with major involvement of tumour necrosis factor (TNF) alpha and the interleukin 23 (IL-23)/interleukin 17A (IL-17A) axis [1, 2, 10, 11]. TNF alpha has been shown to have a substantial impact on osteoclastogenesis and osteoclast activation [12, 13]. The TNF alpha-mediated upregulation of receptors for the nuclear factor kappa beta ligand (RANKL) results in nuclear factor kappa beta signalling pathway enhancement and promotion of osteoclast line differentiation and activation. Furthermore, TNF-alpha contributes to the inhibition of osteoblast differentiation [12, 13]. Osteoclast activation and osteoblast suppression lead to the formation of bone erosions, commonly seen during the course of PsA. On the other hand, other studies revealed that TNF alpha may be also capable of inducing osteoblast differentiation under specific circumstances [14]. TNF alpha level has been shown to be elevated in the synovial fluid and synovial membrane in PsA [15].

Interleukin-17A may contribute to bone destruction in inflammatory rheumatic diseases due to the promotion of RANKL activation, favouring osteoclast-dependent bone resorption [16, 17]. It has also been shown that IL-17A induces synoviocyte activation, migration and invasion in rheumatoid arthritis, as well as matrix metalloproteinase activation, prompting cartilage and matrix destruction, respectively [18]. On the other hand, the pro-osteogenic features of IL-17A have been also well established since IL-17A stimulates the proliferation and differentiation of human mesenchymal stem cells (hMSCs) into osteoblasts. This mechanism is assumed to be associated with the downregulation of DKK1, as shown by Osta et al. [19]. CD8 + T cells producing IL-17A are present in the synovial fluid of inflamed joints in PsA [20].

## Wnt and DKK1 interplay in osteogenesis

### Wnt signalling pathway

One of the major promotors of osteogenesis is the Wingless protein (Wnt) signalling pathway. Wnts are a family of secreted glycoproteins, which exert their activity by binding to a coreceptor complex of frizzled protein (Fzd) and low-density lipoprotein receptor-related protein (LRP) 5 or 6 on osteoblasts and their progenitor cells. This signalling cascade is known as the canonical pathway (versus alternative pathways of Wnt signalling) and leads to a disturbance of the intracellular beta-catenin degradation complex. It results in the accumulation of beta-catenin, which is a key signal transducer of the Wnt canonical pathway, in the cytoplasm and then its translocation to the nucleus, where it regulates gene expression via interaction with transcription factors—T cell factor (TCF)/ Lymphoid enhancer-binding factor (LEF) [21, 22]. The modulated genes are involved in osteoblast proliferation, maturation, terminal differentiation and bone formation. Mature osteoblasts produce osteoprotegerin (OPG), which plays a role as a decoy receptor for the nuclear factor kappa beta ligand (RANKL), thus inhibiting the last stages of osteoclastogenesis [23]. In conclusion, activating the Wnt/beta-catenin signalling pathway strongly promotes osteogenesis.

### DKK1 as Wnt antagonist

Several molecules have been discovered to be Wnt pathway inhibitors [24]. Among them, a soluble protein Dickkopf-related protein 1 (DKK1)—a member of the Dickkopf proteins family—is one of the potent natural suppressors, promoting bone resorption [4, 25]. At first, DKK1 was known to be a key regulator of embryogenesis [26]. Its significance in bone pathological processes was described in human myeloma, in which higher DKK1 levels were observed in the group with osteolytic lesions compared to the group without such pathology [27]. The detailed mechanism of inhibiting Wnt signalling by DKK1 is still the subject of debate; however, it seems that DKK1 binds to LRP 5/6 receptor and triggers the coreceptor complex internalisation [28]. As a consequence, it leads to a decrease in receptor availability for Wnt proteins. Subsequently, this process results in the stabilisation of the beta-catenin degradation complex via phosphorylation, which prevents beta catenin translocation to the nucleus and subsequent gene modulation. These data prove that DKK1 upregulation is negatively correlated with signalling by the Wnt/beta-catenin cascade. The main source of DKK1 in human blood serum is platelets [29, 30].

### Other molecules involved in osteogenesis

Among the other molecules involved in osteogenesis, sclerostin (SOST)—another Wnt pathway antagonist—plays an important role [31]. SOST has been shown to bind to LRP 5 and LRP 6 coreceptor, thus inhibiting Wnt signalling [32]. Low levels of serum sclerostin were associated with syndesmophyte formation in ankylosing spondylitis (AS) [33]. However, the exact role of sclerostin in PsA is not clear. In the study by Fassio et al. [34], sclerostin serum level did not differ between PsA, rheumatoid arthritis (RA) and healthy control groups. Interestingly, in another study performed by this author, sclerostin serum level increased in the PsA group after treatment with secukinumab—anti-IL17A inhibitor [35].

Another relevant signalling pathway responsible for bone formation by promoting osteoblast differentiation is associated with bone morphogenetic proteins (BMPs). These molecules belong to the transforming growth factor beta (TGF beta) superfamily [9, 11]. In the murine model of arthritis, BMP inhibition by the systemic gene transfer of noggin—a BMP antagonist, which exerted preventive as well as therapeutic effects, depending on the time of administration (before or after the first symptoms of arthritis) [36]. In PsA, BMP-7 serum level has been found to be higher than in the control group and correlated with the severity of enthesitis [37]; however, increased serum levels were also reported in AS and RA in another study [38].

## DKK1 in rheumatic diseases

### Animal models

The significance of DKK1 in bone remodelling has been demonstrated in animal models of arthritis. In a mouse model of inflammatory arthritis shown by Diarra et al., the blockade of TNF alpha or DKK1 changed the pattern of damage from osteodestructive to osteoproliferative [5]. In another study by Uderhardt et al., the inhibition of DKK1 in TNF alpha transgenic mice led to the reduced formation of bone erosions and also resulted in ankylosis of the sacroiliac joints [39]. Consistent with this, a recent study demonstrated that the blockade of DKK1 in two mouse models of inflammatory arthritis resulted in significantly elevated periosteal new bone formation. Conversely, transgenic animals with osteoblast-specific overexpression of Dkk1 developed significantly less periosteal new bone compared to their wild-type littermates [40]. Regarding PsA, the PsA-like features have been described in a complex SpA animal model called SKG mice [41]. In this model, skin inflammation and psoriatic skin lesions have been implicated in the dysfunctional IL-23/IL-17 axis, which is also characteristic of human psoriasis. Interestingly, DKK1 levels were significantly elevated in the serum of disease-induced SKG animals [41]. Similarly, serum DKK1 levels were found to be elevated in curdlan-treated SKG mice in a study aimed at deciphering the role of dihydrotestosterone on osteoblast activity in SpA pathogenesis [42]. Importantly, the treatment of curdlan-administered mice with dutasteride (a 5-α reductase inhibitor) reversed the DKK1 levels to the baseline, which resulted in upregulated bone formation in the spine of SKG animals [42].

### Rheumatoid arthritis

Data on the association of DKK1 with bone remodelling are derived not only from animal models but also from clinical studies. In RA, DKK1 serum levels have been determined to be significantly higher than in the control groups [43] and were positively correlated with disease activity and structural damage progression [25, 44, 45]. The association between TNF alpha and DKK1 level was also found, which along with the data indicating a DKK1 decrease during treatment with TNF alpha inhibitors (TNFi) [44], suggests the influence of this cytokine on DKK1 expression in RA. As the main pathological changes in bone remodelling observed in RA are bone erosions, it would be consistent with the known mechanism of Wnt signalling inhibition by DKK1.

### Axial spondyloarthritis/ankylosing spondylitis

On the other hand, data on the relationship between DKK1 expression and the course of disease or various clinical outcomes in the group of SpA remain unclear. There have been a number of studies regarding DKK1 association with bone remodelling in axial spondyloarthritis (axSpA); however, the results are inconsistent. Some reports indicate lower serum DKK1 levels in axSpA or AS subjects compared to healthy controls and RA patients [46–49]. On the contrary, there are some data suggesting no difference or higher serum levels of DKK1 in axSpA/AS [50–53]. Two meta-analyses on this matter were performed, one indicating elevated DKK1 serum levels in AS patients in comparison to healthy controls [54], with the other showing no significant differences between AS and healthy controls [55]. There is also some discrepancy regarding the association of DKK1 serum concentration with clinical activity, structural damage progression, and serum levels of cytokines such as TNF alpha and IL-17A in the course of axSpA/AS.

## Studies on DKK1 serum level in PsA

There are several studies regarding DKK1 serum levels in PsA subjects. The characteristics of selected parameters from these reports are shown in Table 1.

**Table 1:** Basic characteristics and DKK1 serum level in the analysed cohorts

References	PsA pts included (*n*)	Female sex (*n*; %)	Age (years)	Disease duration (years)	Control pts included (*n*)	Method of serum DKK1 measurement	DKK1 level in PsA group*	DKK1 level in comparative group^#^
Daousiss [51]	15	7 (46.6%)	45.4 (± 7.2)	8.0 (4.0–14.0)	45—AS 45—RA 50—HC	DuoSet ELISA kit (R&D Systems)	2.44 ± 0.26 ng/ml	AS (↑) RA ( ↔) HC ( ↔)
Dalbeth [56]	38	16 (42.0%)	50.0 (26.0–68.0)	10.0 (0.5–45.0)	10—PsC 12—HC	ELISA. R&D duoset	2.98 (1.16–5.91) ng/ml	Ps(↓) HC (↓)
Jadon [58]	127 pPsA 117 PsSpA	61 (48.0%)—pPsA 43 (36.7%)—PsSpA	58.5 (50.3–66.6)—pPsA 59.5 (59.6–66.5)—PsSpA	15.0 (7.0–26.0)—pPsA 18.0 (9.0–27.0)—PsSpA	157—AS 200—PsC 50—HC	Quantikine ELISA kits (R&D systems)	3.03 (1.93–3.69) ng/ml—pPsA 3.34 (2.43–4.44) ng/ml -PsSpA	AS + PsSpA (↑)^$^ AS (↑)^&^
Fassio [34]	33	33 (100.0%)	58.8 (± 8.8)	< 3 years (inclusion criterion)	28—RA 35 HC	ELISA (Biomedica)	0.50 ± 0.34 ng/ml	RA (↑) HC (↑)
Fassio [35]	28	18 (64.3%)	57.0 (± 10.0)	< 3 years (inclusion criterion)	43—HC	ELISA (Biomedica)	0.52 ± 0.35 ng/ml	HC (↑)
Diani [59]	50	11 (22.0%)	48.0 (28.0–79.0)	2.1 (0.3–9.2)	50—PsC 20—HC	ELISA (Quantikine, R&D Systems Inc.)	2.80 (2.02–3.53) ng/ml	Ps( ↔) HC (↓)
Chung [60]	69	39 (56.5%)	52.7 (± 13.0)	5.0 (1.6–13.0)	39—RA 21—HC	ELISA kit (Cloud-Clone Corp.)	9.27 ± 3.28 ng/ml	RA (↓) HC (↓)
Abd el Hamid [61]	45	30 (66.7%)	42.2 (± 6.6)	5.0 (1.5–10.0)	45—HC	ELISA kit (Thermo Fischer Scientific)	9.09 (4.89–14.0) ng/ml	HC (↓)

*AS* ankylosing spondylitis, *HC* healthy controls, *pPsA* peripheral psoriatic arthritis, *PsA* psoriatic arthritis, *Ps* psoriasis, *PsSpA* psoriatic axial spondyloarthropathy, *pts* patients, *RA* rheumatoid arthritis

*For better comparison of DKK1 serum levels between studies original units were converted to ng/ml. The original values and units are present in text

^#^The arrows indicate the relative level of DKK1 in comparative group with regard to the PsA group

^$^Compared to pPsA group

^&^Compared to PsSpA group

The cross-sectional study by Daoussis et al. involved AS, RA, PsA patients and controls. Serum DKK1 levels were measured by the ELISA kit. All measurements were performed in triplicate and a mean value was calculated. The DKK1 serum concentration was 2443 ± 255.7 pg/ml in the PsA subgroup. It was lower than in AS subgroup (*p = *0.049 by unpaired *t* test) and similar to that of the RA subgroup and healthy controls (*p = *0.430 by one-way ANOVA). There was no difference in DKK1 values between men and women [51].

In another study performed by Dalbeth et al., there were 38 patients with PsA. Their study assessed several potential biomarkers of bone remodelling. There were 2 control groups—one with patients diagnosed with psoriasis and the other with healthy controls; both included subjects without arthritis. Serum DKK1 level was measured by ELISA kit and was 2977 (1163–5908) pg/ml in the PsA group, which was higher than in control groups (*p* < 0.001 and *p* < 0.01, respectively). The PsA group was also divided into two subgroups—with and without erosions. Both subgroups had higher DKK1 serum concentrations than the psoriasis group. The study did not reveal the relationship between DKK1 levels and distinct bone disease patterns in PsA [56].

A systematic review by Jadon et al., including studies evaluating soluble bone turnover biomarkers in PsA and psoriatic spondyloarthropathy, was published in 2015 [57]. The analysis of DKK1 association with PsA was based only on the one study, discussed above [56].

Another study by Jadon et al. was designed to compare the serum concentrations of several biomarkers, including DKK1, in PsA (divided into subgroups of peripheral psoriatic arthritis (pPsA) and psoriatic axial spondyloarthropathy (PsSpA)), psoriasis, AS and healthy controls. There were 127 pPsA and 117 PsSpA subjects recruited for this study. Serum DKK1 concentrations were measured by ELISA kit. They were 3.03 (1.93–3.69) ng/ml in pPsA and 3.34 (2.43–4.44) ng/ml in PsSpA group and were not statistically different (*p = *0.28; 95% CI = 0.95–1.19). DKK1 serum levels were higher in the combined group of PsSpA and AS (median 3.42 ng/ml; IQR 2.75–4.49) in comparison with pPsA—(adjusted OR = 1.22 per ng/ml increase, 95% CI = 1.05–1.42; *p = *0.01). Additionally, patients with AS had higher DKK1 serum levels than those with PsSpA (adjusted OR = 1.18 per ng/ml increase, 95% CI = 1.05–1.35; *p = *0.02). In further analyses, the panel including DKK1 with metalloproteinase 3 (MMP-3) and osteoprotegerin (OPG) serum levels was the best at distinguishing subjects with PsA from healthy controls. DKK1 level was also shown to be lower in PsSpA subjects with higher Psoriatic Arthritis Spondylitis Radiology Index (PASRI) erosion scores (adjusted OR = 0.28 per ng/ml increase, 95% CI = 0.10–0.80; *p = *0.02). The threshold DKK1 serum concentration, differentiating PsA subjects with or without axial disease was also proposed at the level of 4.96 ng/ml (higher values for axial involvement; sensitivity = 0.15, specificity = 0.94; AUC = 0.56, 95% CI = 0.44–0.67) [58].

Fassio et al. carried out a cross-sectional study of 33 females with PsA using ELISA for serum DKK1 assessment. DKK1 serum levels in the PsA group were 19.32 ± 13.04 pmol/l and were significantly lower in comparison with RA and healthy controls (*p* < 0.01 and *p* < 0.05 by ANOVA test, respectively) [34].

Another study by Fassio et al. has been designed to assess the change in serum biomarker levels in the PsA group following the administration of the anti-IL-17A drug secukinumab utilising an ELISA kit. There were 28 patients in the PsA group and 43 healthy controls. Baseline mean DKK1 levels in the serum of the PsA group were 20.0 ± 13.64 pmol/l, which was significantly lower than in healthy controls (*p* < 0.05). In comparison to the baseline, DKK1 serum concentrations in the PsA group significantly increased after a 6-month treatment with secukinumab (*p* < 0.05) [35].

In the study by Diani et al., biomarkers, including DKK1, were evaluated for the ability to distinguish psoriasis from PsA and healthy controls. The number of patients with PsA, psoriasis and healthy controls was 50, 50 and 20, respectively. DKK1 was measured by the ELISA test. Serum levels of DKK1 in the PsA group were 2.80 (2.02–3.53) ng/ml, which was significantly higher than in healthy controls (*p* < 0.001); however, there was no difference between psoriasis and PsA [59].

Another study by Chung et al. included 69 patients with PsA. Serum DKK1 levels were measured with an ELISA kit. DKK1 concentration was elevated in 68.1% of patients with PsA and was 9.269 ± 3.276 ng/ml. It was higher than in the groups encompassing RA subjects and healthy controls (*p = *0.027; *t* = 2.506 and *p* < 0.010; *t* = 4.323, respectively). The estimated threshold, distinguishing the elevated and normal levels of DKK1, was determined at the level of 7.651 ng/ml. An increase in DKK1 concentration was correlated with elevated swollen joint count (*r* = 0.370; *p* < 0.01), number of platelets (*r* =  − 0.341; *p* < 0.01), C3 (*r* =  − 0.530; *p* < 0.001) and C4 (*r* =  − 0.354; *p* < 0.01). It was postulated that serum DKK1 level is a predictive indicator for bone erosions [60].

Abd el Hamid et al. carried out the case–control study, including 45 PsA subjects. ELISA kit was used for serum DKK1 concentration measurements. Serum DKK1 level was 9090 (4890–14000) pg/ml and was significantly higher than in the control group (*p* < 0.001). Higher DKK1 level was found to be associated with higher disease severity (*p* < 0.001) as well as PsA Disease Activity Score (PASDAS) (*p* < 0.001), Simplified Psoriatic Arthritis Radiographic Score (SPARS) (*p* < 0.001) and PsA Impact of Disease (PsAID) (*p = *0.001) [61].

Wirth et al. prepared a systematic review, regarding various biomarkers associated with diagnosis or prognosis in PsA, including DKK1. The meta-analysis was conducted for studies with serum DKK1 level but it did not show a relevant difference between PsA and healthy controls or psoriasis groups. Of note, the meta-analysis encompassed only two studies—([58, 59]) [62].

Recently, the study by Wahba et al. has been published as corrected proof (article in press). It focused on the correlation of DKK1 serum level with disease acivity and enthesopathy. The final version of this publication is still in progress at the time of submitting our manuscript [63].

## Discussion and conclusions

Psoriatic arthritis is a very heterogenous entity with the concurrent presence of osteoresorption (e.g. erosions) and osteogenesis (e.g. syndesmophytes). It differs from RA, where the balance is significantly shifted towards osteoresorption, and from AS, where excessive bone formation is usually more prominent. One might expect DKK1 expression to be related to the predominant lesions in PsA—higher expression consistent with erosions as a manifestation of excessive osteoresorption and lower expression with entesophytes and syndesmophytes due to excessive osteogenesis.

Nevertheless, the literature review indicates substantial discrepancies between the studies concerning DKK1 serum concentration in PsA. The scarcity of the available data does not allow a reliable meta-analysis to be performed. In 2015, Jadon et al. published a systematic review regarding numerous bone turnover biomarkers in PsA; however, serum DKK1 concentrations were assessed only in one study [57]. In 2022, the review by Tao et al. was released, encompassing studies dealing with the association of DKK1 expression and different autoimmune diseases, including PsA [64]. In another study published in 2022, a meta-analysis of studies with measured serum DKK1 level was performed but it was based only on two papers, which limits the reliable conclusions [62].

Most studies show higher DKK1 serum concentrations in comparison to control groups. Similar to RA, the study by Chung et al. suggested an association of higher DKK1 serum level with the presence of erosions [60]. However, another study by Jadon et al. showed lower DKK1 concentrations in patients with a higher PASRI erosion score [58]. On the other hand, Fassio et al. presented contrasting results indicating lower DKK1 serum levels in PsA subjects [34]. Moreover, these results were supported by another study by Fassio et al., which also showed an increase in DKK1 serum levels after anti-IL17A treatment (secukinumab) [35]. In several randomised controlled trials and one prospective study, this drug proved to be efficient in the reduction of clinical symptoms and inflammation, as well as inhibiting structural damage progression in AS (osteogenesis) and PsA (osteoresorption and osteogenesis) [65–68]. Therefore, the influence of secukinumab on the elevation of DKK1 may suggest that baseline pre-treatment to lower DKK1 could be associated with progressive osteoresorption and osteogenesis in PsA and that IL-17A could also be responsible for DKK1 level modulation, as reflected by low serum DKK1 levels before treatment and higher levels after treatment in the study by Fassio. One recent study was not included in the review, as its final version is still in progress [63].

There are several possible explanations for these inconsistencies. Firstly, the methodology for measuring DKK1 serum levels is not unified. Different ELISA kits have been used in the analysed studies, which may account for the inconsistent results. When the serum DKK1 concentration units were uniformly converted to ng/ml for the purpose of our review, four out of 8 of the discussed studies had comparable DKK1 serum levels [51, 56, 58, 59], two had a remarkably higher level than these four [60, 61] and two were lower [34, 35]. Moreover, the differences were observed when functional serum levels of DKK1 were measured (detected as the capacity to bind LRP-5/6). The dysfunction of DKK1 was also postulated in AS [51], which may explain its higher total serum concentration in some studies [50, 52, 54], while lower levels would be expected based on its function of inhibiting osteogenesis. Such phenomenon might also account for the observations made by Jadon et al., showing that higher DKK1 serum levels correlate with axial disease in the spectrum of SpA [58]. Higher levels may result from the compensative mechanism of DKK1 overexpression due to its impaired function [51, 58].

Secondly, patient groups were frequently small and inconsistent in terms of age, gender proportion, disease activity and duration, as well as different drug usage. Some of the studies also suffer from the lack of important data, regarding group characteristics, e.g. treatment and disease activity.

In addition, the time and drug-related changes of serum DKK1 during the natural history of PsA should be taken into account, which requires subsequent measurements over time. Finally, taking into account the heterogeneity of PsA cases, the distinct subsets of the disease may be marked by different DKK1 expression. Therefore, PsA should probably be subcategorised to assess this issue more thoroughly.

Another emerging issue might be the analysis of DKK1 expression in local tissues like joint synovium. Studies investigating DKK1 expression in synovial tissue and/or synovial fluid were performed in RA and axSpA [69–71]. Recently, the analysis of some biomarkers collected from peripheral joint synovium of PsA patients was published. Among these biomarkers, DKK1 expression was measured and found to be correlated with higher TGF beta expression in the group with erosive PsA [72].

The analysis of local biomarker expression in the area of pathological processes, e.g. synovial inflammation, may provide more unequivocal results regarding its correlation with expected disease outcomes.

There are several limitations of our study. First, the selection bias is possible. Some articles (e.g. not written in English or conference abstracts) were excluded from the analysis, which may affect final conclusions. However, most of the relevant studies are published in English and searching through five comprehensive libraries should limit the possible bias. Secondly, as mentioned before, the cohorts in the selected studies were not homogenous. The different duration of disease or courses of treatment (especially biologic therapy) may significantly influence the results and influence the interpretation of the outcomes. Nevertheless, taking into account relatively small number of the studies, regarding DKK1 level in PsA, their subcategorization is difficult. Probably, it would be feasible, when more data on this issue are available. Moreover, we did not perform the quantitative assessment of the analysed associations in the form of meta-analysis, however, the number of included studies still would not ensure reliable results.

In conclusion, the true relative serum concentration of DKK1 in PsA, in comparison to control groups, as well as its influence on osteogenesis and osteoresorption is still equivocal. Greater number of studies revealed higher DKK1 serum level in PsA than in control groups. Nevertheless, due to small and inhomogeneous cohorts a reliable quantitative assessment cannot be performed. Increase of DKK1 serum level after the treatment (IL-17A inhibitor) has been shown in one study [35], however, contrary to several other studies, baseline DKK1 level was lower in PsA than in the control group. Further research on this matter with consistent and stringent methodology is warranted. Gathering more data of good quality may allow systematic reviews and/or meta-analyses to be constructed to determine the real impact of DKK1 in PsA. Additionally, not only measuring total DKK1 levels but also its functional capacity and local tissue involvement may be beneficial and should be considered in future studies.
